# Enabling C_2_H_2_/CO_2_ Separation Under Humid Conditions with a Methylated Copper MOF

**DOI:** 10.1002/advs.202310025

**Published:** 2024-02-26

**Authors:** Yan‐Long Zhao, Qiancheng Chen, Xin Zhang, Jian‐Rong Li

**Affiliations:** ^1^ Beijing Key Laboratory for Green Catalysis and Separation and Department of Chemical Engineering College of Materials Science & Engineering Beijing University of Technology Beijing 100124 P. R. China

**Keywords:** humidity, linker alkylation, metal‐organic frameworks, open metal site, separation

## Abstract

As a unique subclass of metal‐organic frameworks (MOFs), MOFs with open metal site (OMS) are demonstrated efficient gas separation performance through pi complexation with unsaturated hydrocarbons. However, their practical application faces the challenge of humidity that causes structure degradation and completive binding at the OMS. In this work, the effect of linker methylation of a copper MOF (BUT‐155) on the C_2_H_2_/CO_2_ separation performance under humid condition is evaluated. The water adsorption isotherm, adsorption kinetics, and breakthrough under dry and humid conditions are performed. The BUT‐155 with methylated linker exhibits lower water uptake and adsorption kinetics under humid condition (RH = 20%), in comparison with HKUST‐1. Therefore, the C_2_H_2_/CO_2_ separation performance of BUT‐155 is much less affected by water, especially under higher gas flow rate. Moreover, the dynamic C_2_H_2_/CO_2_ separation performance of BUT‐155 can maintain five breakthrough cycles under humid conditions (RH = 20% and RH = 80%) without obvious performance degradation.

## Graphical abstract

The presence of abundant methyl groups in a Cu‐MOF leads to significantly reduced water uptake and slower water adsorption kinetics, thereby protecting the framework from hydrolysis, as well as inhibiting the water binding at open metal sites. Subsequently, C_2_H_2_/CO_2_ separation under different humid levels is realized with high recyclability.

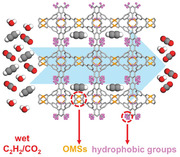

## 1 Introduction

MOFs, a rapidly evolving class of porous materials, have attracted broad attention across numerous fields due to their highly tunable pore size and functionality.^[^ ^1^, ^2^, ^3^, ^4^ ^]^ One such attribute, OMSs or coordinatively unsaturated metal sites, endows MOFs with significant advantages in several critical applications, including catalysis, gas storage, and gas separation.^[^ ^5^ ^]^ For instance, OMSs can selectively adsorb olefins or alkynes through π‐complexation interactions, enabling their purification. Notably, Fe‐MOF‐74 (CPO‐27‐Fe) exhibited exceptional olefin/paraffin separation performance, owing to its Fe(II) sites with high olefin selectivity.^[^ ^6^ ^]^ Besides, the separation selectivity can be improved through tuning the guest binding mode of OMS as demonstrated by UTSA‐74.^[^ ^7^ ^]^ Moreover, OMSs like Cu(I) and Ag(I)can also be incorporated through post‐synthetically functionalization strategies.^[^ ^8^, ^9^ ^]^

Water vapor can significantly influence the stability and separation efficiency of MOFs, particularly those incorporating OMSs, posing a substantial challenge in the practical separation scenarios.^[^ ^5^ ^]^ In terms of stability, most MOFs with OMS exhibit poor water resistance, resulting in the gradual hydrolysis.^[^ ^10^ ^]^ In addition, the high surface tension of water (72.7 mN m^−1^ in 293 K) leads to strong capillary force during water desorption, which is accountable for the pore collapse.^[^ ^11^, ^12^, ^13^ ^]^ Moreover, the competitive binding of water over olefin or acetylene at OMSs could dramatically reduce the adsorption capacity and separation performance. Therefore, strategies for mitigating the impact of water toward MOFs during the separation process are highly desired.

It has been demonstrated that enhancing the hydrophobicity of MOFs effectively protects MOFs from moisture.^[^ ^10^, ^14^ ^]^ For example, coating MOFs with hydrophobic materials such as graphite oxide (GO) ^[^ ^15^ ^]^ and polydimethylsiloxane (PDS) ^[^ ^16^ ^]^ could safeguard MOFs from water attack. Very recently, by coating a hydrophobic COF on moisture‐sensitive MOF‐5 realized significantly improved moisture stability.^[^ ^17^ ^]^ Such a new method improved the coating evenness and facilitated mass transfer given the porous structure of the COF layer. Moreover, the water resistance of MOFs could also be enhanced by introducing hydrophobic molecules, such as imidazole,^[^ ^18^ ^]^ glycine,^[^ ^19^ ^]^ and ionic liquids,^[^ ^20^ ^]^ onto the OMS. Among these strategies, the direct linker hydrophobic modification with alkyl or fluoroalkyl groups could ensure the structural uniformity and avoid the OMS occupation. Currently, construction of hydrophobic pore environment in MOFs through ligand design has been proven to be an effective approach to promote the MOF stability, and enabled C_2_H_6_/C_2_H_4_,^[^ ^21^ ^]^ C_3_H_8_/C_3_H_6_^[^ ^22^ ^]^ and CO_2_/N_2_^[^ ^23^, ^24^ ^]^ separation under humid conditions. Due to the hydrophilic nature of OMS, it is interesting to know if linker alkylation is capable to enhance the water resistance of MOFs with OMS, which has rarely been explored. Moreover, such assessment is of high significance toward the practical application of this unique class of MOFs.

The purification of C_2_H_2_ from CO_2_ is an important chemical separation process that has been realized by MOFs‐based adsorbents.^[^ ^25^, ^26^, ^27^, ^28^, ^29^, ^30^, ^31^ ^]^ Herein, we investigated a methylated Cu‐MOFs (BUT‐155) for C_2_H_2_/CO_2_ separation under humid conditions. BUT‐155 demonstrates high C_2_H_2_ adsorption capacity (145.1 cm^3^ g^−1^ at 298 K and 1 bar) and commendable C_2_H_2_/CO_2_ selectivity (6.43 at 298 K and 1 bar). In comparison with a typical Cu‐MOF with OMS, the methylated ligand imparts BUT‐155 enhanced hydrophobicity, thereby dramatically prohibiting water adsorption under low humidity. Moreover, the water adsorption kinetics is also lower, which synergistically reduce the water interference during the separation process. Only a slight reduction (≈10%) of the C_2_H_2_ dynamic adsorption was observed for BUT‐155. Besides, BUT‐155 exhibited excellent C_2_H_2_/CO_2_ separation recyclability (>5 cycles) under various humidity levels (RH = 20% or 80%). In contrast, the dynamic C_2_H_2_ capacity of HKUST‐1 decreases by ≈45% after five breakthrough cycles even at low humidity (RH = 20%). This study demonstrates that the adverse effect of water can be significantly diminished by introduction of alkylated linkers for MOFs with OMSs, thus promoting their separation performance under humid conditions.

## 2 Experimental Section

### 2.1 Materials and Physical Measurements

All solvents (AR grade) and reagents were commercially available and directly used without further purification. Powder X‐ray diffraction patterns were recorded on a Bruker D8‐Focus Bragg‐Brentano X‐ray powder diffractometer equipped with a Cu sealed tube (*λ* = 1.54178 Å) at room temperature (RT). The single‐component gas sorption measurements were performed on Micrometrics ASAP 2020 surface area analyzer. Kinetic adsorption was measured with the BSD‐DVS dynamic gas sorption analyzer. The experiments temperatures were controlled by liquid nitrogen bath (77 K), ice‐water bath (273 K), and water bath (298 K) respectively.

### 2.2 Synthesis and Activation of MOFs

HKUST‐1 were synthesized and activated according to the literature methods.^[^ ^32^ ^]^ The ligand of BUT‐155 (H_8_tdhb) was synthesized by following previous literature while the crystal of BUT‐155 was synthesized with slight modification.^[^ ^33^ ^]^ CuCl_2_·2H_2_O (0.1 mmol), H_8_tdhb (0.01 mmol), and 2 mL HBF_4_ (40 wt%) were ultrasonically dissolved in 10 mL of DMF in a 20 mL Pyrex vial. The vial was sealed and then heated at 80 °C overnight in an oven. After cooling to room temperature, blue rod‐shaped crystals were collected by filtration and washed with DMF to remove the unreacted ligand and salt. The as‐synthesized sample of BUT‐155 was soaked in DMF for 72 h during which fresh DMF was exchanged for six times and then soaked in methanol for 48 h during which fresh methanol was exchanged for four times. Before gas adsorption experiments, the sample of MOFs was activated at 120 °C for 15 h.

### 2.3 Breakthrough Experiments

The breakthrough experiments were carried out in a self‐made dynamic mixed‐gas breakthrough setup. A stainless‐steel column with inner dimensions of ϕ = 4 × 80 mm was used for sample packing and both ends were filled with silica glass wool. The MOFs adsorbent was activated in situ for 18 h under 100 °C and high vacuum, and then a helium flow (20 mL min^−1^) was introduced upon cooling to room temperature. The mixed‐gas flow and pressure were controlled by using a pressure control valve and a mass flow controller. Outlet effluent from the column was continuously monitored using gas chromatography with a thermal conductivity detector (TCD). The mixed‐gas flow rate during the breakthrough process was 2 and 4 mL min^−1^ using the C_2_H_2_/CO_2_ (1/1, v/v) mixture at room temperature (298 K). After the breakthrough experiment, the desorption curve was measured under He flow of 20 mL min^−1^ at 100 °C. Between each breakthrough cycle (under dry/wet feed gas), the sample was regenerated by vacuum pumping for 6 h at 100 °C, followed by the He flow (20 mL min^−1^) for 2 h at 100 °C. The gas adsorption capacity in column can be determined by Equation (1):

(1) $$q_{i}=\;\frac{C_{i}V}{22.4\times m}\;\times \;\int_{0}^{t}\left(1-\frac{F}{F_{0}}\right)dt$$

In Equation (1), *q_i_* (mmol g^−1^) is the adsorption capacity of component *i*, *C_i_* is the mole fraction of component *i* in the feed gas, *V* (mL min^−1^) is the flow rate of feed gas, *t* (min) is the adsorption time, *m* (g) is the mass of the adsorbent in column, *F* and *F*_0_ are the outlet and inlet flow rates (mL min^−1^) of component *i*, respectively.

## 3 Results and Discussion

**Figure 1:**
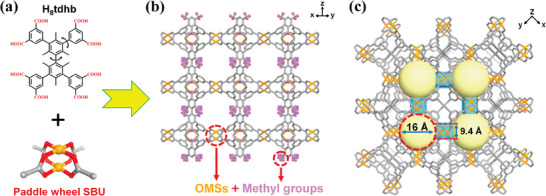
a) Structure of the ligands and paddle wheel SBU of BUT‐155. b) Schematic diagram of pore environment with the highlight of rich methyl groups (pink) and OMSs (yellow). c) The crystal structure of BUT‐155 in view of c‐axis, exhibiting the cuboctahedron cages with the diameter of 16 Å and the channel aperture of 9.4 Å.

The microporous BUT‐155 was synthesized under solvothermal conditions.^[^ ^24^ ^]^ The phase purity was confirmed by comparing the powder X‐ray diffraction (PXRD) patterns and simulated patterns derived from the crystal structure (Figure S1a, Supporting Information). BUT‐155 is constructed from the classic Cu‐paddlewheel SBU and tdhb^8−^ with eight carboxyl groups wherein each neighboring phenyl rings in the tdhb^8−^ ligand are ideally perpendicular due to the steric hindrance induced by methyl groups (**Figure** **1**a). The structure of BUT‐155 is composed of tightly arranged cuboctahedron cages with a diameter of ≈16 Å and aperture of 9.4 Å (Figure 1c; Figure S4, Supporting Information). There are abundant OMSs within the channel that adorned with methyl groups (Figure 1b). The OMSs are potential binding sites for preferential C_2_H_2_ adsorption over CO_2_. ^[^ ^34^ ^]^ Besides, the high‐density methyl groups in BUT‐155 are crucial for stabilizing the framework against water through enhancing the rigidity and hydrophobicity of BUT‐155.^[^ ^14^, ^35^ ^]^ The abundant OMSs and high‐density methyl groups in the framework make it an ideal prototype MOF to study the gas separation under humid conditions.

**Figure 2:**
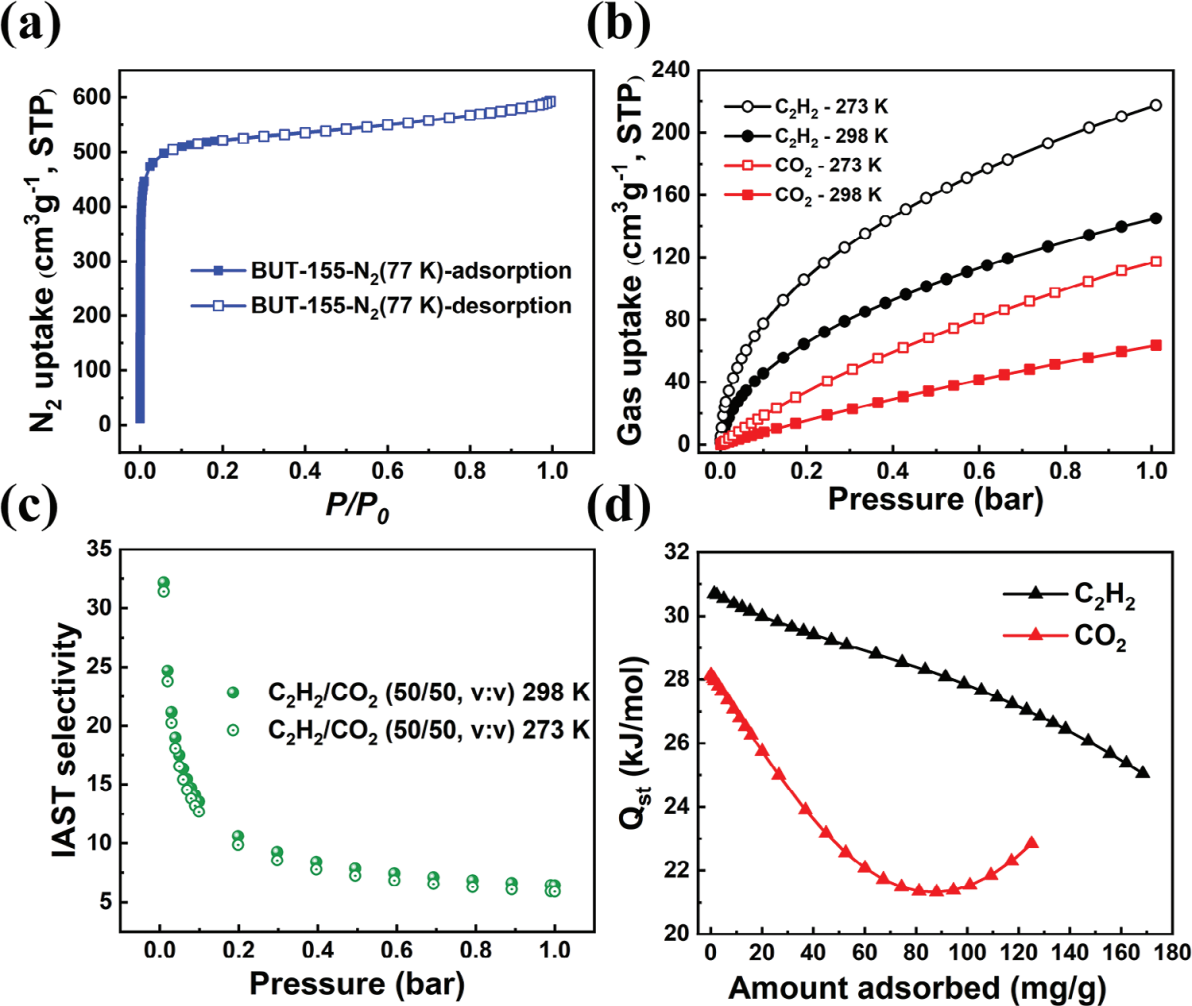
a) N2 adsorption at 77 K of BUT‐155. b) C2H2 and CO2 adsorption isotherms of BUT‐155 at 298 and 273 K. c) C2H2/CO2 (50/50) IAST selectivity at 298 and 273 K of BUT‐155. d) Qst of C2H2 and CO2 for BUT‐155.

As depicted in **Figure** **2**a, the permanent porosity of BUT‐155 was established via N_2_ adsorption at 77 K. The Brunauer–Emmett–Teller (BET) surface area was calculated to be 2097 m^2^ g^−1^, accompanied by a total pore volume of 0.91 cm^3^ g^−1^, being comparable to reported values (2070 m^2^ g^−1^ and 0.820 cm^3^ g^−1^). The C_2_H_2_ and CO_2_ adsorption isotherms for BUT‐155 showed preferential adsorption of C_2_H_2_ over CO_2_ (Figure 2b). The C_2_H_2_ adsorption capacities at 1 bar are 145.1 and 217.6 cm^3^ g^−1^ at 298 and 273 K, respectively, which are ≈2.28 (298 K) and 1.85 (273 K) times those of CO_2_ adsorption capacity. Such adsorption preference is prevalent in MOFs containing Cu‐paddle wheel SBUs, and can be attributed to π‐complexation between OMSs and alkynes.^[^ ^36^, ^37^, ^38^ ^]^ Furthermore, the C_2_H_2_/CO_2_ selectivity (50:50, v/v) was determined using the ideal adsorbed solution theory (IAST) (Figures S5–S8, Supporting Information). As shown in Figure 2c, the C_2_H_2_/CO_2_ selectivity ranges from 32.17 to 6.43 at 298 K, being comparable to or higher than typical MOFs with Cu paddle‐wheel units (Table S3, Supporting Information). The isoteric heats of adsorption (Qst) values for C_2_H_2_ and CO_2_ were calculated to be 30.7 and 28.1 kJ mol^−1^ at zero coverage, respectively by using Virial method (Figure 2d; Figures S9 and S10, Supporting Information), indicating the stronger binding interaction toward C_2_H_2_. Besides, the density functional theory (DFT) calculations were also carried out to identify the adsorption sites of C_2_H_2_ and CO_2_ (Figures S11–S13 and Tables S1 and S2, Supporting Information). As anticipated, OMS from the paddle wheel structure in BUT‐155 serves as the primary C_2_H_2_ adsorption site through π‐complexation interaction, accompanied by multiple C‐H···O interactions (Figure S11b, Supporting Information). The CO_2_ exhibits relatively weak O···Cu interaction with OMS (Figure S11a, Supporting Information).

**Figure 3:**
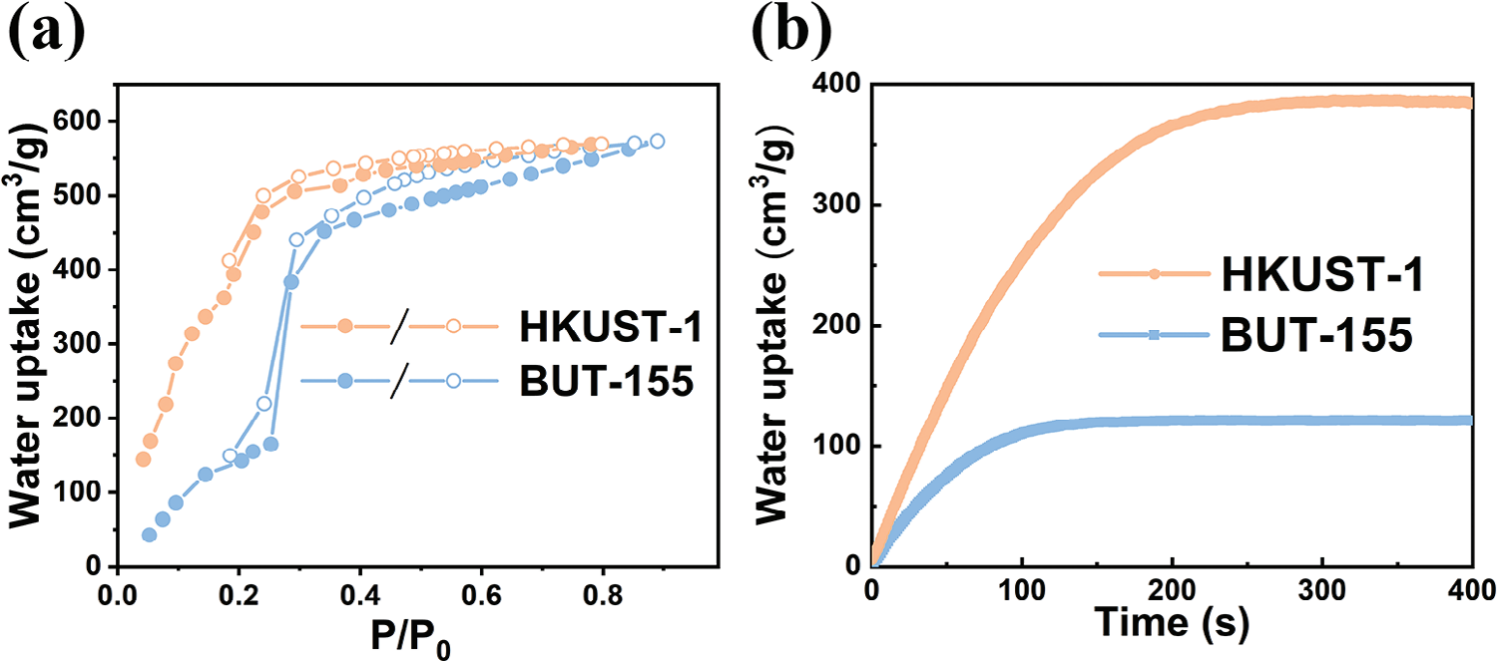
a) Water vapor adsorption isotherms of BUT‐155 and HKUST‐1 at 25 °C. b) Water adsorption kinetics of BUT‐155 and HKUST‐1 under RH = 20% at 298 K.

In addition to the commendable C_2_H_2_ capacity and C_2_H_2_/CO_2_ selectivity, water tolerance is crucial in industrial separation process. Therefore, the water affinity of BUT‐155 was further examined. To examine the impact of ligand methylation on water adsorption of MOFs, the classic HKUST‐1 with a methyl‐free ligand and a similar pore volume (0.78 cm^3^ g^−1^) was selected for comparison. As shown in **Figure** **3**a, although the total water adsorption capacities of these two MOFs are comparable, HKUST‐1 exhibits high water adsorption at low pressure and almost reach saturation under P/P*_0_* = 0.3, likely due to its relatively hydrophilic pore surface with rich OMSs.^[^ ^10^, ^33^ ^]^ In contrast, BUT‐155 did not exhibits much water uptake until the P/P*_0_* reach 0.25, indicating its lower affinity for water, despite the presence of hydrophilic OMSs in the framework. This could be attributed to the introduction of high‐density methyl groups that promoted the hydrophobicity of BUT‐155. Moreover, the water adsorption kinetics was also investigated. As shown in Figure 3b, the equilibrium water uptakes of HKUST‐1 (≈385 cm^3^ g^−1^) and BUT‐155 (≈121 cm^3^ g^−1^) correspond well to their water adsorption capacity at P/P*_0_* = 0.2, respectively. And the slope of kinetic adsorption curve of HKUST‐1 is notably higher than that of BUT‐155, indicating a faster water adsorption rate (Figure 3b). Furthermore, the initial adsorption rate of HKUST‐1 was found to be 1.87 times that of BUT‐155 (Figures S14 and S15, Supporting Information). Such results further suggest that the enhanced pore surface hydrophobicity could also reduce the water adsorption kinetics, therefore inhibit water adsorption from both thermodynamic and kinetic aspects.

**Figure 4:**
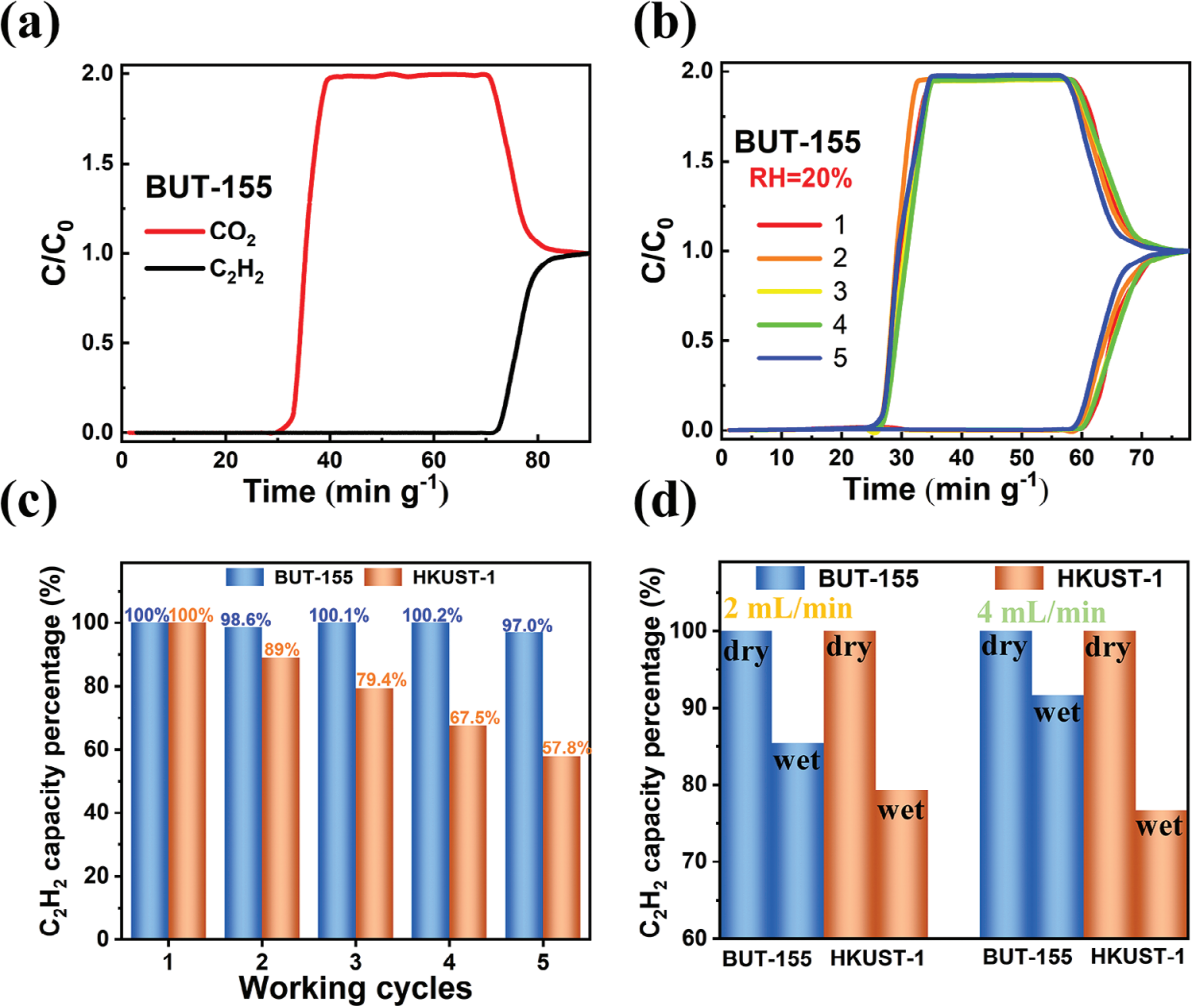
a) Breakthrough separation of C2H2/CO2 (50/50) mixture at flow rate of 2 mL min^−1^ with BUT‐155. b) Five cycles of breakthrough separation of C2H2/CO2 (50/50) mixture at flow rate of 2 mL min^−1^ under RH = 20% with BUT‐155. c) Retention of acetylene adsorption of BUT‐155 and HKUST‐1 in the five cycles of breakthrough experiments of C2H2/CO2 (50/50) mixture under RH=20%. d) Comparison of the acetylene dynamic adsorption of BUT‐155 and HKUST‐1 under dry and RH=20% humidity, and the effect of flow rates.

The reduced water uptake and kinetics of BUT‐155 motivated us to further evaluate the C_2_H_2_/CO_2_ dynamic separation under humid conditions. The column breakthrough experiment was first carried out at 298 K with an equimolar C_2_H_2_/CO_2_ (50/50, v/v) dry mixture under a flow rate of 2 mL min^−1^. As depicted in **Figure** **4**a, the separation of C_2_H_2_ from equimolar gas mixture could be effectively realized by BUT‐155 where CO_2_ broke through the column first at 31 min g^−1^, followed by the breakthrough of C_2_H_2_ at 72 min g^−1^. The breakthrough interval is ≈41 min g^−1^, surpassing benchmark materials such as FJU‐90 (22 min g^−1^),^[^ ^39^ ^]^ ZJU‐74 (36 min g^−1^),^[^ ^40^ ^]^ UTSA‐300 (30 min g^−1^),^[^ ^41^ ^]^ and JNU‐2 (23 min g^−1^).^[^ ^42^ ^]^ The dynamic C_2_H_2_ capacity of BUT‐155 was calculated as 76.4 cm^3^ g^−1^ by integration of the breakthrough curve.^[^ ^43^ ^]^ Besides, 30.8 mL g^−1^ C_2_H_2_ with purity >98.93% could be obtained from the desorption process of breakthrough experiment (Figure S16a, Supporting Information). Moreover, after four additional repetitions of re‐activation and breakthrough experiments, the curves are largely consistent with the initial one (Figure S16b, Supporting Information). The PXRD pattern of BUT‐155 sample after the cyclic experiments remained the same (Figure S1a, Supporting Information), indicating the high durability of BUT‐155 during the C_2_H_2_/CO_2_ separation process. Subsequently, the breakthrough experiments were then conducted under RH = 20% humidity conditions. Under this condition, BUT‐155 continued to maintain five breakthrough cycles without significant decline in separation performance, albeit with the slightly earlier C_2_H_2_ breakthrough time compared to the dry feed gas condition. The C_2_H_2_ adsorption capacity under humid conditions was calculated as 65.3 cm^3^ g^−1^, 85.5% of the capacity for dry gas (Figure 4b; Figure S18a, Supporting Information). The decrease in C_2_H_2_ adsorption capacity under humid condition could be attributed to the competitive adsorption between C_2_H_2_ and H_2_O on the hydrophilic OMS. Nevertheless, the coordinated water will not cause damage to the structure of BUT‐155 and could be removed for subsequent breakthrough cycle under re‐activation. Additionally, cyclic breakthrough experiments under 80% RH further suggest the excellent recyclability of BUT‐155 under high humidity (Figure S18b, Supporting Information). Furthermore, it is evidenced that BUT‐155 not only remains stable under moisture gas, but also maintains integrity even in water treatment for 24 h, according to the N_2_ uptake at 77 K (Figure S2, Supporting Information). For comparison, the breakthrough experiments were also conducted on HKUST‐1. Under dry feed gas, HKSUT‐1 barely maintained 5 cycles of breakthrough cycles with a total degradation of ≈10% (Figure S17, Supporting Information). Once the humidity is introduced, the performance degradation became more significant. HKUST‐1 gradually lose the separation performance for each breakthrough cycle under humidity, and eventually exhibited ≈45% and 73% performance degradation under 20% and 80% RH after five cycles, respectively (Figure S19, Supporting Information). Besides, the morphological of BUT‐155 remains almost the same before and after the breakthrough experiments in humid condition although crystal cracking was observed, while the shape of HKUST‐1 changes from polyhedron to strip (Figure S3, Supporting Information). These evidences corroborate the role of hydrophobicity in enhancing recyclability under humid conditions (Figure 4c).

Moreover, although the dynamic selectivity of both BUT‐155 and HKUST‐1 decreased after introducing humidity (Figures S20 and S21, Supporting Information), the reduction percentage of C_2_H_2_ uptake caused by humidity for BUT‐155 (14.5%) is lower than that in HKUST‐1 (20.7%), which might be explained from multiple perspectives. From the water adsorption thermodynamics perspective, BUT‐155 exhibits less water uptake at 20% RH compared to HKUST‐1, resulting in relatively more available OMSs for C_2_H_2_ uptake in BUT‐155 (Figure 3a). From the water adsorption kinetics perspective, the water diffusion rate in BUT‐155 is slower, hindering its competitive binding at OMSs with C_2_H_2_. To further validate this hypothesis, the breakthrough experiments with higher flow rate (4 mL min^−1^) were performed (Figure S22, Supporting Information). The dynamic C_2_H_2_ capacities of BUT‐155 were calculated as 72.6 and 66.5 cm^3^ g^−1^ under the dry and wet (RH = 20%) conditions, respectively. The higher flow rate indeed decreased the influence of water, only 8.4% decrease of dynamic C_2_H_2_ capacity. In contrast, water adsorption of HKUST‐1 is less affected by the increasing flow rate, as the water adsorption could be rapidly achieved (Figure 4d), likely due to the faster water diffusion in HKUST‐1. On the other hand, the slower water diffusion in BUT‐155 results in less water uptake at higher flow rates, providing more OMSs for C_2_H_2_ binding. Overall, we can conclude that alkylation of pore surface offers efficient protection to OMSs against water through reduced water uptake capacity and kinetics under low humidity, thus maintaining the separation performance to a higher extent.

## 4 Conclusion

In summary, BUT‐155 exhibits high C_2_H_2_ adsorption capacity (145.1 cm^3^ g^−1^ at 298 K and 1 bar) and commendable C_2_H_2_/CO_2_ selectivity (6.43 at 298 K and 1 bar) thanks to the presence of abundant OMSs. The high‐density methyl groups enhanced the hydrophobicity of BUT‐155, thus inhibiting water adsorption from both thermodynamic and kinetic aspects. In the breakthrough experiments under humid condition (RH = 20%), the separation performance of BUT‐155 is much less affected by water in comparison with HKUST‐1, especially under higher flow rate. Moreover, the dynamic C_2_H_2_/CO_2_ separation performance of BUT‐155 could maintain five breakthrough cycles under humid condition (RH = 20% and RH = 80%) without degradation. These results demonstrated linker alkylation is an efficient approach to enhance the hydrophobicity of MOF with OMS, thus paving the way to utilize this unique type of MOFs for broader separation applications under humid conditions.

## Conflict of Interest

The authors declare no conflict of interest.

## Supporting information

Supporting Information

## Data Availability

The data that support the findings of this study are available from the corresponding author upon reasonable request.
