# Supplementary material for: Enabling C2H2/CO2 Separation Under Humid Conditions with a Methylated Copper MOF
Source: Adv Sci (Weinh). 2024 Feb 26;11(17):2310025. doi: 10.1002/advs.202310025 (PMC11077691; doi:10.1002/advs.202310025)
Supplement: Supplementary file 1 — Supporting Information [file ADVS-11-2310025-s001.pdf]

ADVANCED SCIENCE
Open Access

Supporting Information

for *Adv. Sci.*, DOI 10.1002/advs.202310025

Enabling C_2H_2/CO_2 Separation Under Humid Conditions with a Methylated Copper MOF

Yan-Long Zhao, Qiancheng Chen, Xin Zhang and Jian-Rong Li**

Supporting Information

for

Enabling C_2H_2/CO_2 Separation Under Humid Conditions with a Methylated Copper MOF

Yan-Long Zhao, Qian-Cheng Chen, Xin Zhang and Jian-Rong Li**

Beijing Key Laboratory for Green Catalysis and Separation and Department of Chemical Engineering, College of Materials Science & Engineering, Beijing University of Technology, Beijing 100124, PR China

Email addresses of the corresponding authors: *zhang.xin@bjut.edu.cn (X. Zhang)* and *jrli@bjut.edu.cn (J.-R. Li)*

Supplementary figures and tables:

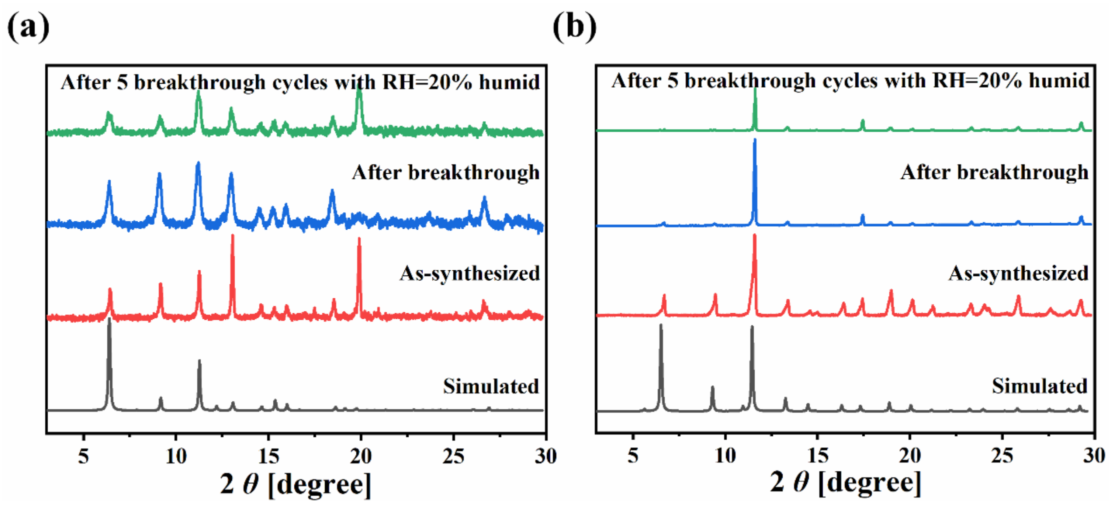

Figure S1. PXRD patterns of (a) BUT-155 and (b) HKUST-1 after breakthrough experiments.

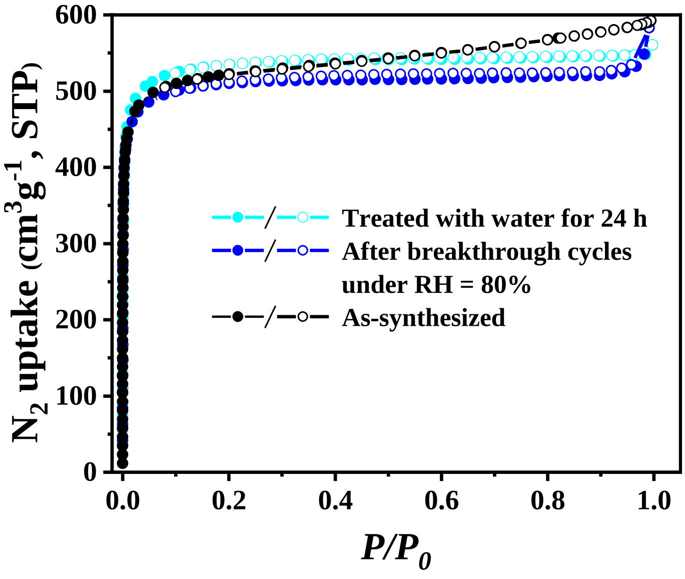

Figure S2. N_2 adsorption/desorption curves at 77 K for BUT-155 after water treatment and breakthrough cycles.

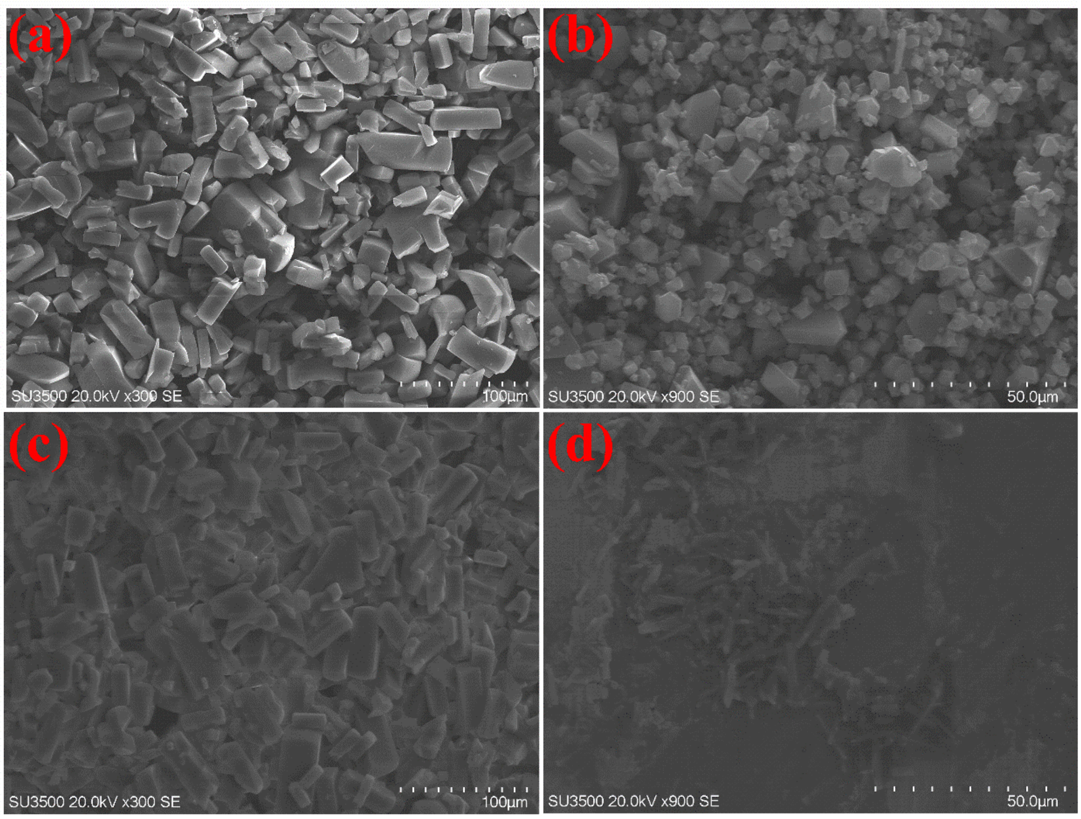

Figure S3. SEM images of as-synthesized (a) BUT-155 and (b) HKUST-1, respectively; SEM images of (c) BUT-155 and (d) HKUST-1 after breakthrough experiments under humid condition.

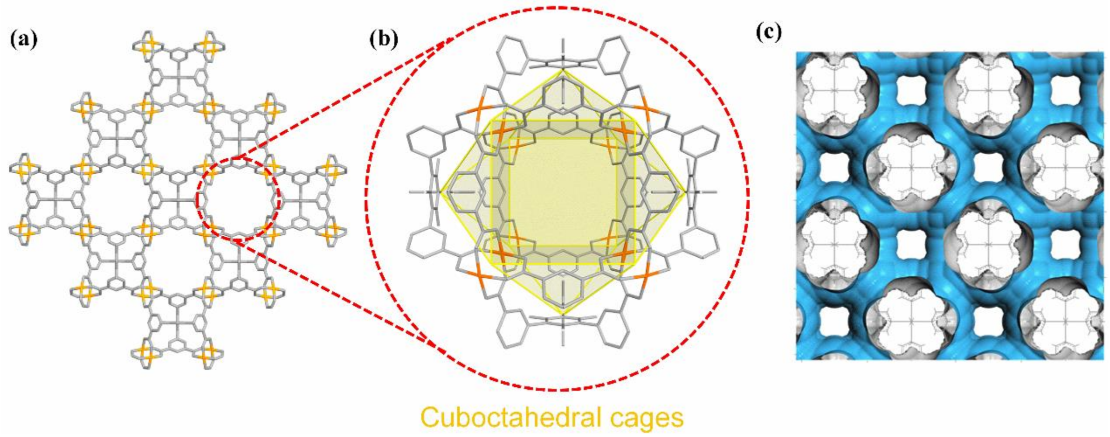

Figure S4. (a) Crystal structure of BUT-155 with the highlight of (b) Cuboctahedral cages; (b) Connolly surface of channel in BUT-155.

Calculation of selectivity via ideal adsorption solution theory (IAST)

The Ideal Adsorbed Solution Theory (IAST) was applied to predict the adsorption selectivity [1]. For a binary mixture of species *i* and *j*, the adsorption selectivity, S_{ads}, is defined as:

$$S_{ads} = \frac{q_i/q_j}{p_i/p_j}$$

Where q represents mole fraction in the mixture and p represents partial pressure at adsorption equilibrium.

The single-component adsorption isotherms of C_2H_2 and CO_2 in BUT-155 were fitted by using single-site Langmuir-Freundlich model:

$$N = A_1 \times \frac{b_1 P^{c_1}}{1+b_1 P^{c_1}} + A_2 \times \frac{b_2 P^{c_2}}{1+b_2 P^{c_2}} \quad (A_2 = 0\text{, single-site model})$$

In this model, N is the adsorption amount in mmol/g, P is the pressure of bulk gas at equilibrium with the adsorbed phase (101 kPa), A_1 and A_2 are the saturation capacities of site A and site B, respectively. And b_1 and b_2 are the affinity coefficients of site A and site B, respectively. C_1 and C_2 represent the deviations from an ideal homogeneous surface. The fitted curves and corresponding parameter values are presented in the following figures (Figure S3-S6)

(a)

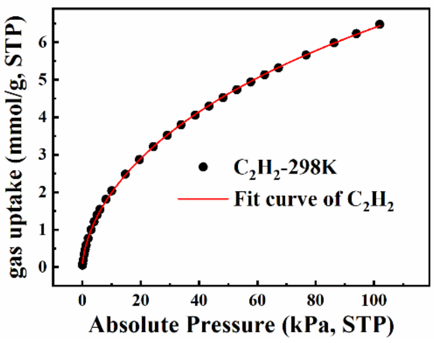

(b)

Equation	A1*b1*x^c1/(1+b1*x^c1)
A1	23.21283 ± 2.05871
b1	0.02308 ± 0.00173
c1	0.60796 ± 0.01037
Adj. R-Square	0.99969

Figure S5. (a) Single-site Langmuir-Freundlich fitting for the C_2H_2 adsorption isotherm of BUT-155 at 298 K. (b) Single-site Langmuir-Freundlich parameters from the fitting of C_2H_2 at 298 K.

(a)

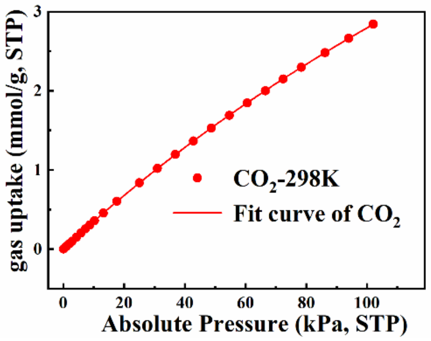

(b)

Equation	A1*b1*x^c1/(1+b1*x^c1)
A1	13.5767 ± 0.2771
b1	0.00269 ± 3.72454E-5
c1	0.99222 ± 0.00274
Adj. R-Square	0.99999

Figure S6. (a) Single-site Langmuir-Freundlich fitting for the CO_2 adsorption isotherm of BUT-155 at 298 K. (b) Single-site Langmuir-Freundlich parameters from the fitting of CO_2 at 298 K.

(a)

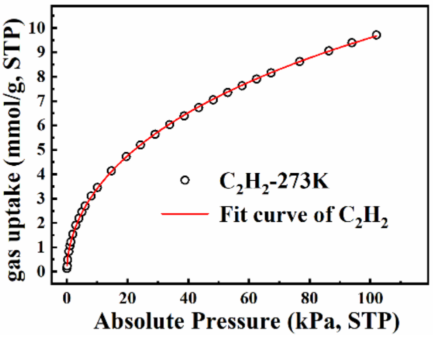

(b)

Equation	A1*b1*x^c1/(1+b1*x^c1)
A1	36.47846 ± 3.07454
b1	0.02978 ± 0.00226
c1	0.53941 ± 0.00832
Adj. R-Square	0.99975

Figure S7. (a) Single-site Langmuir-Freundlich fitting for the C_2H_2 adsorption isotherm of BUT-155 at 273 K. (b) Single-site Langmuir-Freundlich parameters from the fitting of C_2H_2 at 273 K.

(a)

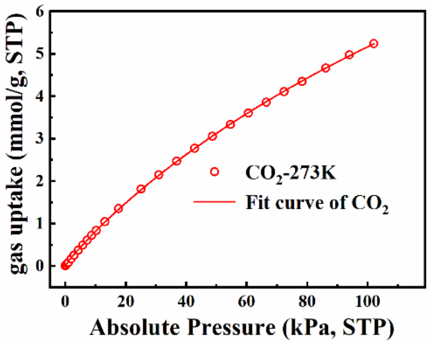

(b)

Equation	A1*b1*x^c1/(1+b1*x^c1)
A1	19.28984 ± 0.82326
b1	0.00531 ± 1.44859E-4
c1	0.91905 ± 0.00726
Adj. R-Square	0.99995

Figure S8. (a) Single-site Langmuir-Freundlich fitting for the CO_2 adsorption isotherm of BUT-155 at 273 K. (b) Single-site Langmuir-Freundlich parameters from the fitting of CO_2 at 273 K.

Isosteric heat of adsorption calculation.

The isosteric enthalpy of adsorption for C_2H_2 and CO_2 were calculated by employing virial equation comprising of the temperature-independent parameters a_i and b_j, where the adsorption data were collected at 273 K and 298 K [2].

$$\ln(P) = \ln(N) + \left(\frac{1}{T}\right)\sum_{i=0}^{m} a_i \times N^i + \sum_{j=0}^{n} b_j \times N^j$$

In the virial equation, N is gas uptake (mg/g), P is pressure (mmHg), a_i and b_j are virial coefficients, m and n are number of coefficients require to adequately describe the isotherm. The parameters that obtained from the fitting of the C_2H_2 and CO_2 adsorption isotherms are presented in Figure S7-8. All isotherms were fitted with $R^2>0.999$.

The obtained parameters were used to calculated the Q_{st} (kJ/mol) within the adsorption capacity range through the virial equation, defined as

$$Q_{st} = -R\sum_{i=0}^{m} a_i N^i$$

Where T is the temperature (K), R is the gas constant (8.314 J/(K·mol)).

(a)

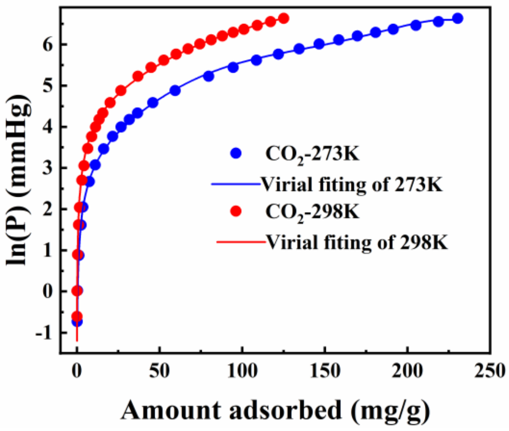

(b)

Equation	ln(x)+1/K*(a0+a1*x+a2*x^2+a3*x^3+a4*x^4+a5*x^5)+(b0+b1*x+b2*x^2)	
a0	-3386.61703± 218.93303	-3386.61703± 218.93303
a1	14.29601 ± 10.96731	14.29601 ± 10.96731
a2	0.0414 ± 0.10153	0.0414 ± 0.10153
a3	-0.00171 ± 6.88231E-4	-0.00171 ± 6.88231E-4
a4	8.00155E-6 ± 3.57616E-6	8.00155E-6 ± 3.57616E-6
a5	-1.3335E-8 ± 6.50332E-9	-1.3335E-8 ± 6.50332E-9
b0	13.1834 ± 0.76212	13.1834 ± 0.76212
b1	-0.0683 ± 0.03828	-0.0683 ± 0.03828
b2	4.0774E-4 ± 3.16041E-4	4.0774E-4 ± 3.16041E-4
K	273 ± 0	298 ± 0

Figure S9. (a) Virial fitting of the C_2H_2 adsorption isotherms at 273 K and 298 K for BUT-155 for Qst calculation. (b) Parameter fits for C_2H_2 adsorption isotherms of BUT-155 using Virial method.

(a)

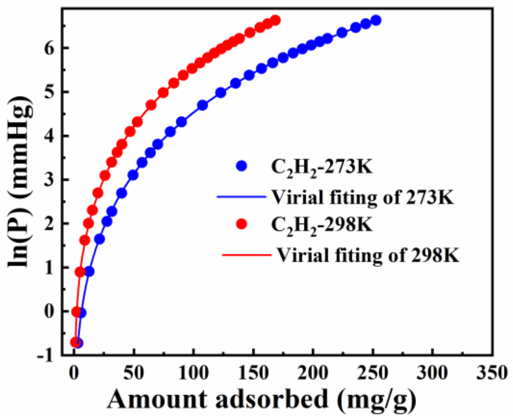

(b)

Equation	ln(x)+1/K*(a0+a1*x+a2*x^2+a3*x^3+a4*x^4+a5*x^5)+(b0+b1*x+b2*x^2)	
a0	-3700.21236 ± 47.53537	-3700.21236 ± 47.53537
a1	5.50896 ± 1.39777	5.50896 ± 1.39777
a2	-0.05217 ± 0.01036	-0.05217 ± 0.01036
a3	4.50094E-4 ± 8.49008E-5	4.50094E-4 ± 8.49008E-5
a4	-1.44744E-6 ± 3.88582E-7	-1.44744E-6 ± 3.88582E-7
a5	1.87301E-9 ± 6.30952E-10	1.87301E-9 ± 6.30952E-10
b0	11.49708 ± 0.16446	11.49708 ± 0.16446
b1	0.01603 ± 0.00483	0.01603 ± 0.00483
b2	-9.13789E-5 ± 2.77343E-5	-9.13789E-5 ± 2.77343E-5
K	273 ± 0	298 ± 0

Figure S10. (a) Virial fitting of the CO_2 adsorption isotherms at 273 K and 298 K for BUT-155 for Qst calculation. (b) Parameter fits for CO_2 adsorption isotherms of BUT-155 using Virial method.

Computational details

Density functional theory (DFT) calculations were performed with Dmol3 module implemented in the Materials Studio software. A double numerical polarized (DNP) basis set was employed to expand the wave functions with an orbital cutoff of 5.2 Å. For the electron–electron exchange and correlation interactions, the generalized gradient approximation (GGA) with Perdew-Burke-Ernzerhof (PBE) exchange-correlation were utilized [3, 4]. The adsorption site was calculated by using the locate task and Metropolis method with 2.0×10^7 equilibration steps, followed by 2.0×10^7 production steps for calculating the ensemble averages. The gas molecules and MOF skeleton were both treated as rigid bodies. The gas-framework interaction and the gas-gas interaction were described by the standard universal force field (UFF). And the atomic partial charges of the framework were used for Qeq method.

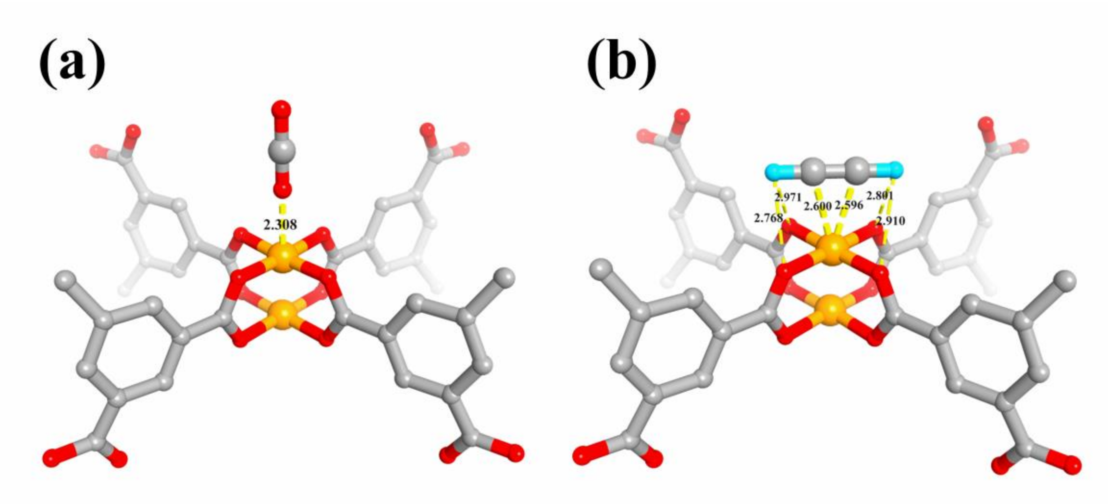

Figure S11. The calculated prior adsorption sites of (a) CO_2 and (b) C_2H_2 in BUT-155.

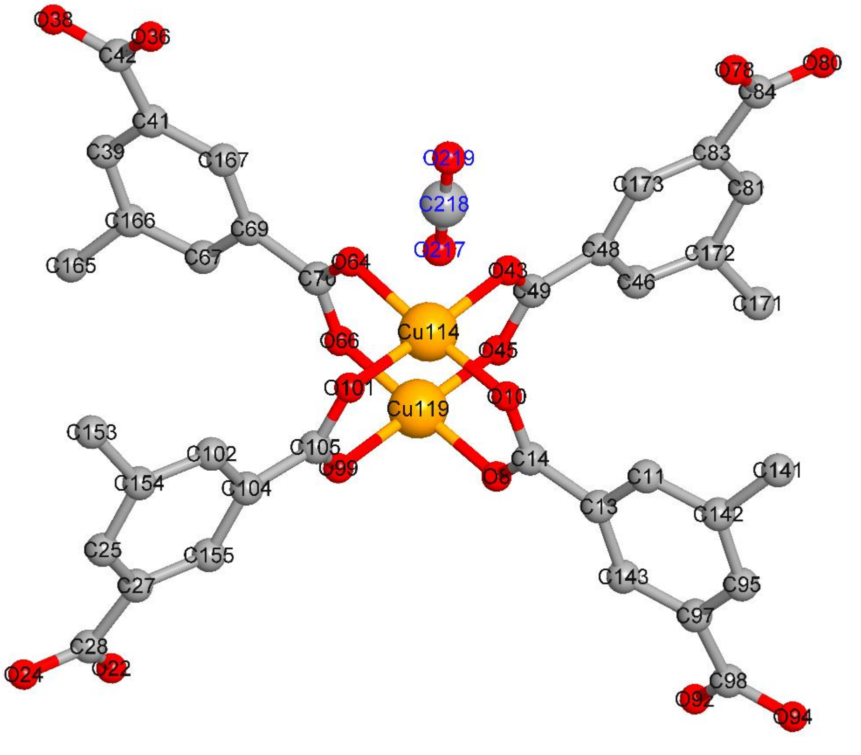

Figure S12. The atomic numbers in the model of CO_2@BUT-155.

Table S1. The calculated atom charges in the model of CO_2@BUT-155

Atom	Charge (e)	Atom	Charge (e)	Atom	Charge (e)
C11	-0.155	C97	-0.017	O22	-0.758
C13	-0.018	C98	0.351	O24	-0.757
C14	0.352	C102	-0.149	O36	-0.758
C25	-0.151	C104	-0.017	O38	-0.757
C27	-0.019	C105	0.352	O43	-0.759
C28	0.351	C141	0.018	O45	-0.757
C39	-0.155	C142	0.351	O64	-0.757
C41	-0.018	C143	-0.176	O66	-0.758
C42	0.351	C153	0.018	O78	-0.759
C46	-0.147	C154	-0.0067	O80	-0.757
C48	-0.017	C155	-0.176	O92	-0.758
C49	0.351	C165	0.019	O94	-0.757
C67	-0.143	C166	-0.00296	O99	-0.758
C69	-0.017	C167	-0.176	O101	-0.757
C70	0.351	C171	0.0188	Cu114	2.66
C81	-0.151	C172	-0.00275	Cu119	2.66
C83	-0.019	C173	-0.176	C218	0.581
C84	0.351	O8	-0.758	O217	-0.291
C95	-0.142	O10	-0.757	O219	-0.291

Table S2. The calculated atom charges in the model of C_2H_2@BUT-155

Atom	Charge (e)	Atom	Charge (e)	Atom	Charge (e)
C4	-0.142	C91	0.351	O29	-0.758
C6	-0.0174	C109	-0.142	O31	-0.757
C7	0.351	C111	-0.0172	O50	-0.759
C18	-0.155	C112	0.351	O52	-0.757
C20	-0.0181	C135	0.0196	O57	-0.759
C21	0.351	C136	-0.00261	O59	-0.757
C32	-0.151	C137	-0.176	O71	-0.758
C34	-0.0183	C147	0.0186	O73	-0.757
C35	0.351	C148	-0.00249	O85	-0.758
C53	-0.155	C149	-0.176	O87	-0.757
C55	-0.0182	C159	0.0187	O106	-0.759
C56	0.351	C160	-0.00293	O108	-0.757
C60	-0.146	C161	-0.176	Cu113	2.66
C62	-0.0171	C177	0.0187	Cu120	2.66
C63	0.351	C178	-0.00296	C217	-0.28
C74	-0.143	C179	-0.176	C218	-0.28
C76	-0.0171	O1	-0.759	H219	0.28
C77	0.351	O3	-0.757	H220	0.28
C88	-0.155	O15	-0.758		
C90	-0.0184	O17	-0.757		

Water adsorption kinetics comparation.

To quantify the difference in water adsorption rates between BUT-155 and HKUST-1, initial adsorption rate (R_0) is employed. The calculation and fitting methods are according to previous literature [5]. Mono-exponential approximation method was selected as the appropriate model to fit the water adsorption behavior of BUT-155 and HKUST-1 with $R^2 > 99\%$ for all fits. The fitting formula and fitting curves are shown in Fig. S14-S15.

The calculation process is as below:

$$\boldsymbol{R = \frac{dM}{dt}}$$

In such equation, *M* represent the adsorption amount (g/g). *t* represents the time (s). *R* represents the adsorption rate (g/g·s).

$$\boldsymbol{R = \frac{dM}{dt}}$$

$$\boldsymbol{R = \frac{dM}{dt} = \frac{d(A \times (1 - e^{-\frac{t}{t_0}})}{dt} = \frac{A}{t_0} \times e^{-\frac{t}{t_0}}}$$

When *t* approaches 0, then:

$$\boldsymbol{R_0 = \frac{A}{t_0}}$$

Herein, the initial adsorption rate comparison between HKUST-1 and can be simplified as:

$$\boldsymbol{\frac{R_{0_{HKUST-1}}}{R_{0_{BUT-155}}} = \frac{\frac{A_{HKUST-1}}{t_{0_{HKUST-1}}}}{\frac{A_{BUT-155}}{t_{0_{BUT-155}}}} = 1.87}$$

(a)

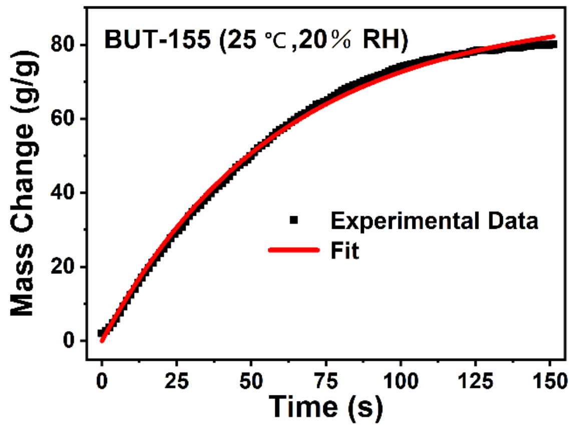

(b)

Model	monoexp
Equation	A*(1-exp(-t/t0))
A	89.23256 ± 0.39649
t0	59.52649 ± 0.6181
Adj. R-Square	0.99788
Reduced Chi-Sqr	1.14549

Figure S14. Mono-exponential approximation of the dynamic water adsorption process in BUT-155 at 25 °C and 20% relative humidity (RH).

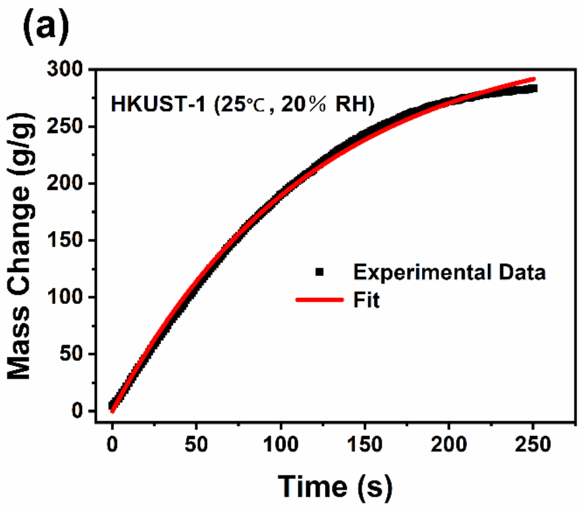

(b)

Model	monoexp
Equation	A*(1-exp(-t/t0))
A	331.85706 ± 1.42428
t0	118.50655 ± 1.05518
Adj. R-Square	0.99788
Reduced Chi-Sqr	1.14549

Figure S15. Mono-exponential approximation of the dynamic water adsorption process in HKUST-1 at 25 °C and 20% relative humidity (RH).

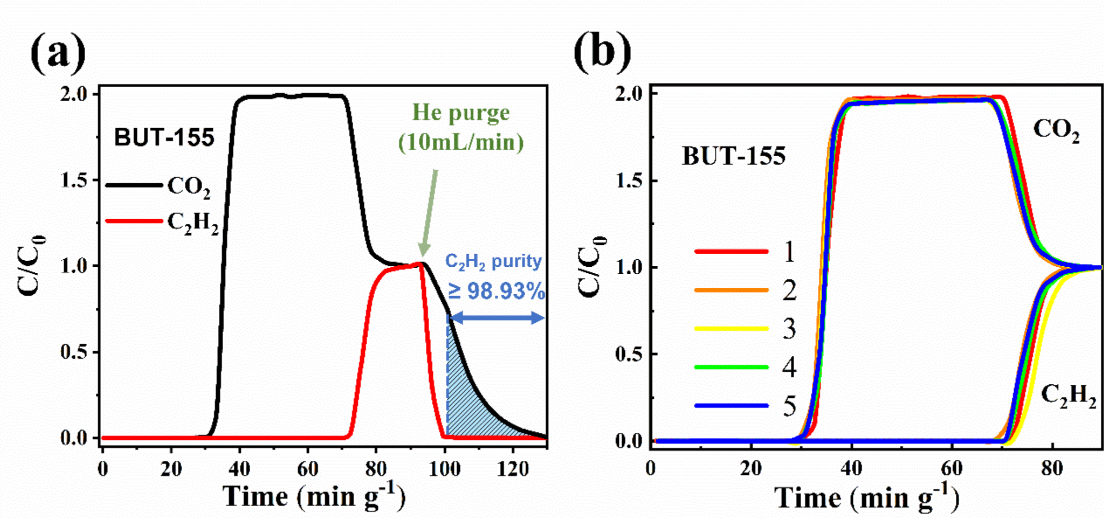

Figure S16. (a) Breakthrough curves for a 50/50 C_2H_2/CO_2 mixture (2 mL/min) on BUT-155 and desorption curves under helium sweeping (10 mL/min) at 298K. (b) Five cycles of breakthrough separation experiments.

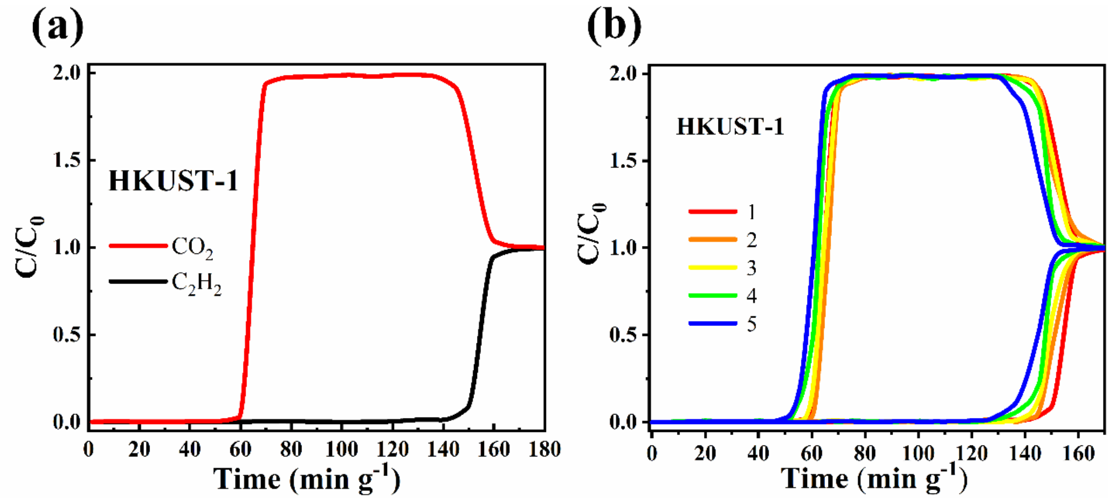

Figure S17. (a) Breakthrough curves for a 50/50 C_2H_2/CO_2 mixture (2 mL/min) on HKUST-1 at 298K. (b) Five cycles of breakthrough separation experiments.

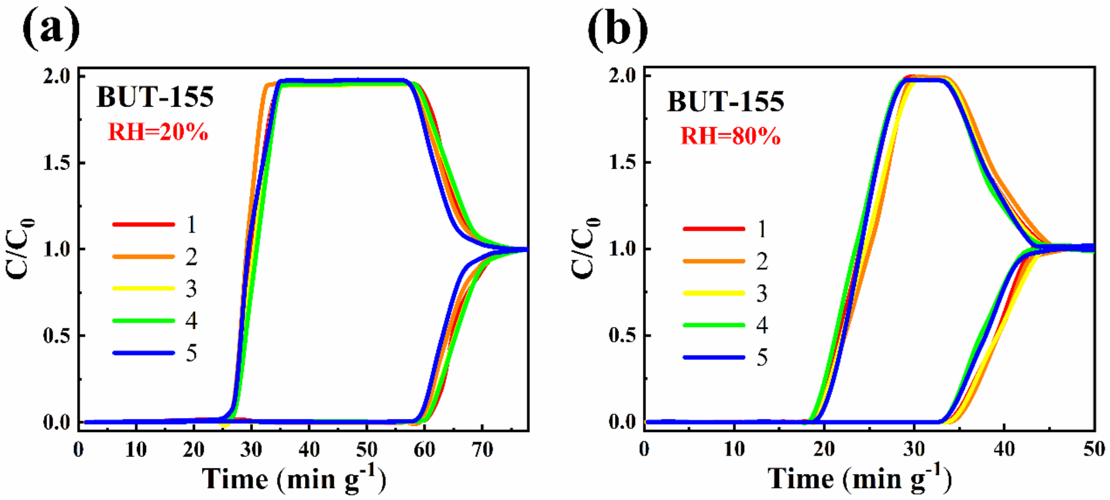

Figure S18. Five cycles of breakthrough separation experiments on BUT-155 for C_2H_2/CO_2 (50/50) mixture at flow rate of 2.0 mL/min under (a) RH = 20% and (b) RH = 80%.

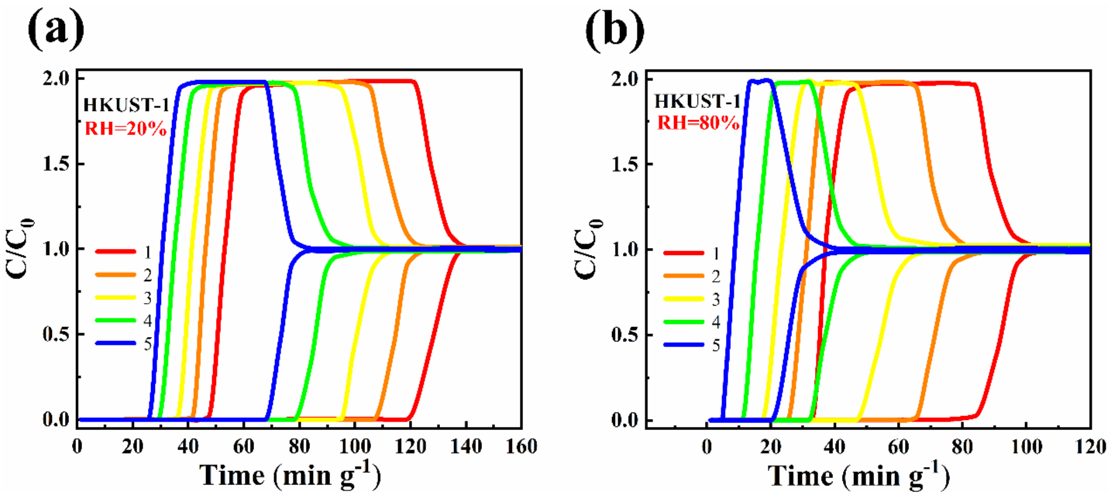

Figure S19. Five cycles of breakthrough separation experiments on HKUST-1 for C_2H_2/CO_2 (50/50) mixture at flow rate of 2.0 mL/min under (a) RH = 20% and (b) RH = 80%.

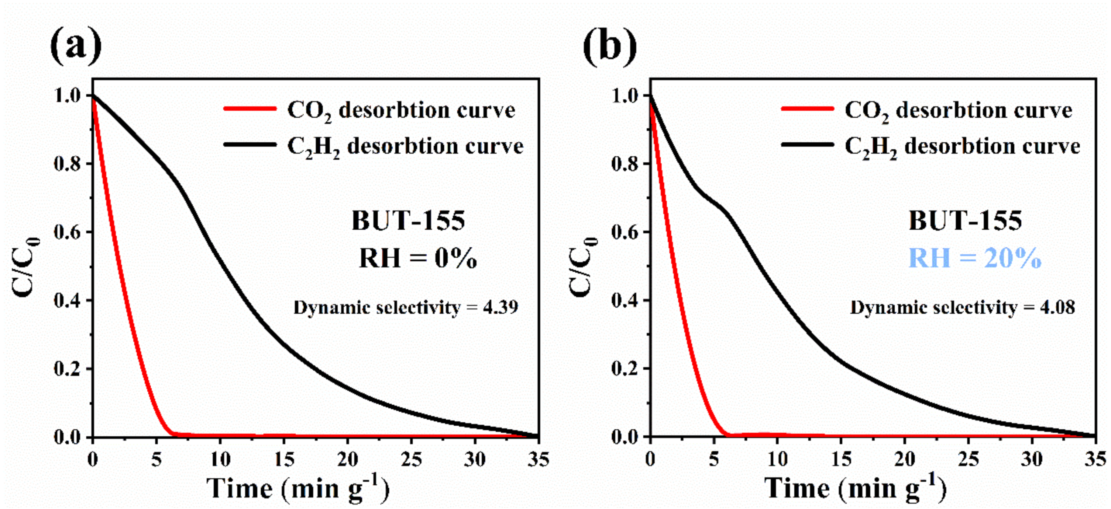

Figure S20. Desorption experiment for BUT-155 after the breakthrough experiment under (a) dry and (b) RH = 20% conditions. The dynamic selectivity was calculated by the ratio the integration of C_2H_2 desorption curve and CO_2 desorption curve.

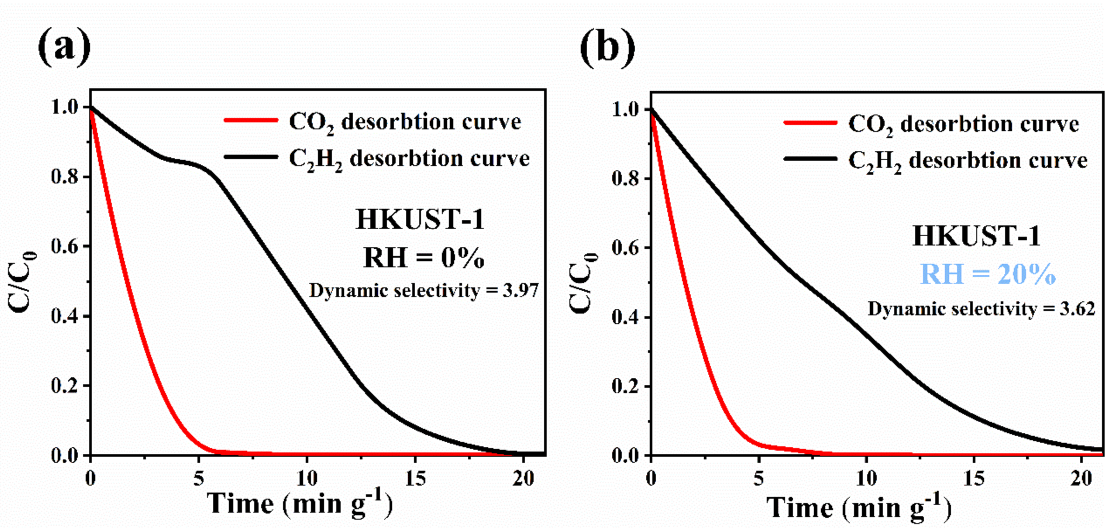

Figure S21. Desorption experiment for HKUST-1 after the breakthrough experiment under (a) dry and (b) RH = 20% conditions. The dynamic selectivity was calculated by the ratio the integration of C_2H_2 desorption curve and CO_2 desorption curve.

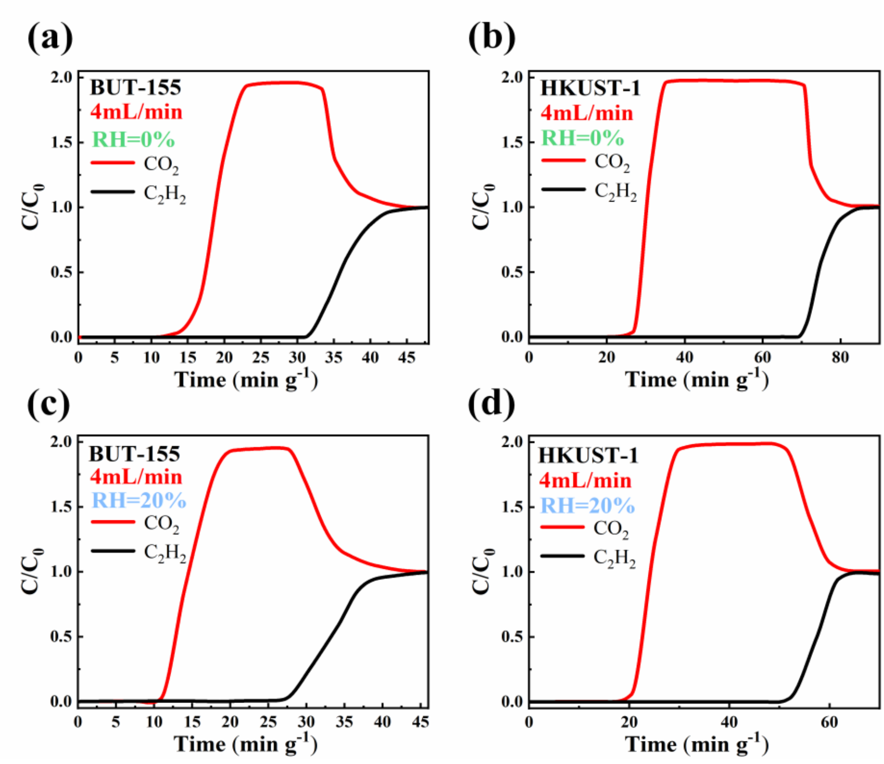

Figure S22. Breakthrough curves for 50/50 C_2H_2/CO_2 mixture (4 mL/min) on (a) BUT-155 and (b) HKUST-1; Breakthrough curves for 50/50 C_2H_2/CO_2 mixture (4 mL/min) under RH = 20% on (c) BUT-155 and (d) HKUST-1.

Table S3. The C_2H_2 and CO_2 adsorption capacity and C_2H_2/CO_2 separation selectivity

of MOFs with Cu-paddle wheel structures.

MOF	Density (g/cm^3)	C_2H_2 uptake (cm^3/g)/(cm^3/cm^3)	CO_2 uptake (cm^3/g)/(cm^3/cm^3)	C_2H_2/CO_2 selectivity (v/v, 1/1)	Qst of C_2H_2/CO_2 (kJ/mol)	Ref.
FJI-H8-Me	0.822	229/188	106/87	5.6	33.7/21.77	[6]
FJI-H8-Et	0.834	217/181	102/85	5.7	-	[6]
FJI-H8-^{i}Pr	0.842	179/151	92/77	5.3	-	[6]
FJI-H8-^{n}Pr	0.842	174/147	90/76	6.0	-	[6]
HKUST-1	1.01	198/200	138/139	2.4	34.8/27.3	[7]
ZJU-50a	1.08	192/207	100/108	12	40/30	[7]
ATC-Cu	1.461	112/164	90/131	53.6	79.1/36	[8]
FJU-H33	0.75	154/116	80/60	4.8	34.3/24.2	[9]
NTU-54	1.413	23/32.5	20/28	3.8	38/35	[10]
NTU-55	0.875	135/118	71/62	4.0	25.3/22	[11]
ZJU-199	0.911	128/117	62/56	4.0	38.5/29.0	[12]
Cu-CPAH	1.317	134/176	89/117	3.6	35.4/31.5	[13]
Cu(BDC-Br)	1.297	34/44	23/30	3.9	26.1/25.6	[14]
**BUT-155**	0.845	145/123	64/54	6.4	30.7/28.1	This work

References

[1] A.L. Myers, J.M. Prausnitz, AIChE J. 1965, 11, 121.
[2] L. Czepirski, J. JagieŁŁo, Chem. Eng. Sci. 1989, 44, 797.
[3] J. P. Perdew, M. Ernzerhof, K. Burke, Rationale for mixing exact exchange with density functional approximations, J. Chem. Phy. 1996, 105, 9982.
[4] J. P. Perdew, K. Burke, M. Ernzerhof, Phys. Rev. Lett. 1996, 77, 3865.
[5] N. Hanikel, M. S. Prévot, F. Fathieh, E. A. Kapustin, H. Lyu, H. Wang, N. J. Diercks, T. G. Glover, O. M. Yaghi, ACS Cent. Sci. 2019, 5, 1699.
[6] Z. Di, C. Liu, J. Pang, C. Chen, F. Hu, D. Yuan, M. Wu, M. Hong, Angew. Chem. Int. Ed. 2021, 60, 10828.
[7] K. Shao, H.M. Wen, C.C. Liang, X. Xiao, X.W. Gu, B. Chen, G. Qian, B. Li, Angew. Chem. Int. Ed. 2022, 61, e202211523.
[8] Z. Niu, X. Cui, T. Pham, G. Verma, P.C. Lan, C. Shan, H. Xing, K.A. Forrest, S. Suepaul, B. Space, A. Nafady, A.M. Al-Enizi, S. Ma, Angew. Chem. Int. Ed. 2021, 60, 5283.
[9] H. Li, C. Chen, Z. Di, Y. Liu, Z. Ji, S. Zou, M. Wu, M. Hong, ACS Appl. Mater. Interfaces 2022, 14, 52216.
[10] S. Liu, Y. Huang, Q. Dong, H. Wang, J. Duan, Inorg. Chem. 2020, 59, 9569.
[11] Q. Dong, Y. Guo, H. Cao, S. Wang, R. Matsuda, J. Duan, ACS Appl. Mater. Interfaces 2020, 12, 3764.
[12] L. Zhang, C. Zou, M. Zhao, K. Jiang, R. Lin, Y. He, C.-D. Wu, Y. Cui, B. Chen, G. Qian, Cryst. Growth Des. 2016, 16, 7194.
[13] L. Meng, L. Yang, C. Chen, X. Dong, S. Ren, G. Li, Y. Li, Y. Han, Z. Shi, S. Feng, ACS Appl. Mater. Interfaces 2020, 12, 5999.
[14] H. Cui, Y. Ye, H. Arman, Z. Li, A. Alsalme, R.-B. Lin, B. Chen, Cryst. Growth Des. 2019, 19, 5829.
